# Increased number of crosslinking sites for factor XIII counteracts the mechanical and lytic destabilization of fibrin caused by citrullination of fibrinogen

**DOI:** 10.3389/fimmu.2026.1897298

**Published:** 2026-07-27

**Authors:** Kornélia Guzmits, Erzsébet Komorowicz, Olivér Ozohanics, Krasimir Kolev, Anna Tanka-Salamon

**Affiliations:** Department of Biochemistry, Institute of Biochemistry and Molecular Biology, Semmelweis University, Budapest, Hungary

**Keywords:** citrullination, crosslinking, fibrinogen, fibrinolysis, FXIII, PAD4

## Abstract

**Introduction:**

In thrombi, fibrin formation is accompanied by neutrophil activation, which leads to the release of neutrophil extracellular traps (NETs). Citrullination is a post-translational modification of arginine catalyzed by peptidylarginine deiminases, which are released during NET formation and modify extracellular proteins. Citrullinated fibrin has been detected within thrombi, but its impact on clot stabilization by activated Factor XIII (FXIIIa) has not been investigated.

**Objective:**

To characterize the effect of FXIIIa on the mechanical strength and lytic susceptibility of citrullinated fibrin.

**Methods:**

FXIII cross-linking sites in fibrin were identified by mass spectrometry. Fibrinolysis was investigated by turbidimetry and viscoelastometry in clots containing plasminogen and tissue plasminogen activator (tPA). Mechanical and viscoelastic parameters of the same clots were determined by oscillatory rheometry and viscoelastometry.

**Results:**

Non-crosslinked fibrin formed from citrullinated fibrinogen showed lower stability in the applied biomechanical and fibrinolytic assays. However, citrullination increased the number of heterochain crosslinks formed by FXIIIa from 27 to 36, and that of homochain crosslinks from 29 to 30. In line with this finding, the antifibrinolytic effect of FXIII crosslinking was consistently more pronounced in citrullinated fibrin. While FXIIIa increased the maximal clot firmness of native fibrin by 10% in the viscoelastometric assay, this FXIII-related rise in mechanical strength was 38% in citrullinated fibrin. Similarly, in the rheometric assays, the FXIII-associated increment in the critical shear stress required to disassemble the clot was higher in citrullinated fibrin than in the native one.

**Conclusion:**

FXIIIa is essential to counteract the weakening effects of citrullination in fibrin clots.

## Graphical Abstract

**Figure:**
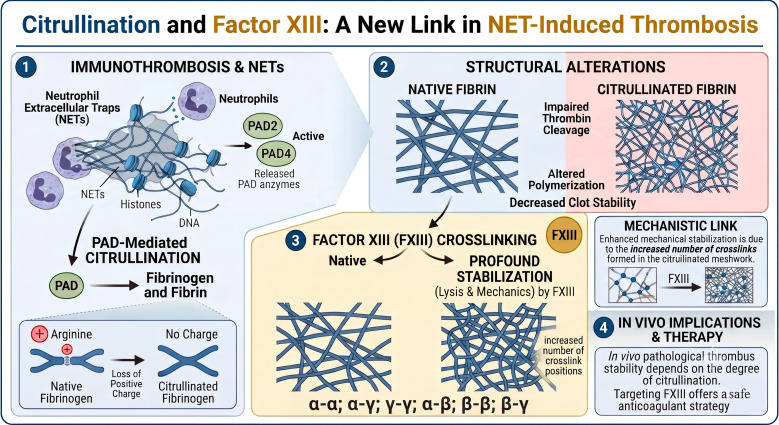
Citrullination and factor XIII: A new link in NET-induced thrombosis.

## 1 Introduction

Neutrophil extracellular traps (NETs) have become a focal point in immunothrombosis research, as growing evidence indicates their role in promoting pro-inflammatory and procoagulant processes. Uncontrolled NET formation has been linked to thrombosis and other cardiovascular complications in both arterial and venous systems (1–4). The hallmark of NETosis is neutrophil DNA decondensation, which, in many cases, is preceded by histone citrullination by peptidyl arginine deiminase-4 (PAD4) (5, 6). Citrullination is a posttranslational modification, that converts positively charged arginine residues into uncharged citrullines, leading to chromatin decondensation. Notably, a recent study reveals that, in addition to histones, PAD4 can citrullinate thousands of intracellular proteins responsible for various cellular functions (7). Extracellular effects of PADs may also play a role, as cytoskeletal rearrangement followed by rupture of the nuclear envelope and ultimately the cell membrane during NET formation allows the release of PADs (PAD2 and 4) into the extracellular space (8, 9). Furthermore, activated neutrophils expose PAD4 on their cell surface and continuously release PAD2 to the extracellular space (10). The main structural determinant of thrombi, fibrin(ogen) is a target molecule of extracellular PADs (11–14). Given the colocalization of activated neutrophils, NETs, and evolving thrombi in immunothrombosis, it is plausible to investigate the impact of fibrinogen citrullination on the stability of the resulting thrombus. *In vivo* data provide evidence for the presence of citrullinated fibrin(ogen) in murine deep vein thrombi, with consequent alterations in the structure and function of the forming clot (15). Densely packed thin fibers are formed from citrullinated fibrinogen resulting in mechanically fragile thrombi with low porosity and high proteolytic resistance (13, 15, 16). Thrombi are further stabilized by activated factor XIII (FXIIIa), a transglutaminase, that forms γ-glutamyl-ϵ-lysyl crosslinks within fibrin, resulting in enhanced mechanical and fibrinolytic resistance (17, 18). Both pH and ionic strength are known to substantially influence the intermolecular forces between fibrin(ogen) molecules (19). It is reasonable to propose that citrullination, by reducing the net charge of fibrinogen, diminishes electrostatic repulsion between molecules, which may promote closer alignment of fibrin fibrils and facilitate the formation of FXIII-mediated crosslinks. The influence of citrullination on fibrin cross-linking by FXIIIa remains contradictory (20, 21). In this study, we aimed to investigate potential cross-linking sites in the structure of citrullinated fibrin and the lytic and mechanical stability of these clots after cross-linking by FXIIIa.

## 2 Materials and methods

### 2.1 Materials

Human fibrinogen, peptidyl-arginine-deiminase 4, streptokinase, and chloride-amidine were purchased from Merck Life Sciences Kft. (Budapest, Hungary). Chromogenic substrate for plasmin Spectrozyme-PL (H-D-norleucyl-hexahydrotyrosyl-lysine-p-nitroanilide) was from BioMedica Diagnostics (Windsor, NS Canada). Bovine thrombin and dithiothreitol (DTT) were from Serva Electrophoresis GmbH (Heidelberg, Germany). Thrombin was further purified by ion-exchange chromatography on sulfopropyl-Sephadex yielding preparation with specific activity of 1200 IU/mg (22) and 1 IU/ml was considered equivalent to approximately 10.7 nM by active site titration (23). Recombinant FXIIIA_2_ and FXIII inhibitor ZED1301 (Ac-(D)-Asp-MA-Nle-Nle-Leu-Pro-Trp-Pro-OH) were from Zedira GmbH, (Darmstadt, Germany). Recombinant tissue-type plasminogen activator (tPA) was the product of Boehringer Ingelheim (Ingelheim am Rhein, Germany). Plasminogen was prepared and activated to plasmin by streptokinase (24, 25). If not otherwise indicated, all experiments were performed in HEPES buffer (10 mM HEPES, 150 mM NaCl, pH 7.4).

### 2.2 Citrullination of fibrinogen

Fibrinogen (9.1 μM) was treated with PAD4 at 270, 620 or 800 ng/mL for 1.5 hours at 37 °C in the presence of 6.8 mM CaCl_2_ and 0.68 mM DTT to achieve low-, medium- and high levels of citrullination. To eliminate the effect of FXIII contamination in the fibrinogen preparation, an FXIII inhibitor (20 µM ZED1301) was included. The citrullination reaction was stopped with 60 µg/mL chloride-amidine. The fibrinogen solution was then dialysed against HEPES buffer. The degree of fibrinogen citrullination was 1) indirectly estimated from the coagulability of fibrinogen, which depends on the availability of arginine at position 16 on the Aα chain and position 14 on the Bβ chain (20, 26) for cleavage by thrombin, and 2) quantitatively characterised by mass spectrometry (MS). For the turbidimetric assays, thrombin (10 nM) was added to citrullinated or native fibrinogen (7.5 μM) in a 96-well microtiter plate in the presence of CaCl_2_ (5 mM). The formation of the fibrin mesh was monitored by measuring the light absorbance at 340 nm with a CLARIOstar spectrophotometer (BMG Labtech, Ortenberg, Germany). The reduction in the A_max_ value of the clotting curve was proportional to the concentration of PAD (**Supplementary Figure 1**). Medium-citrullinated fibrin was selected for further studies.

### 2.3 Mass spectrometry

MS was performed to determine the number of potential crosslinking sites in fibrin and to investigate citrullination events in fibrinogen. Native and citrullinated fibrinogen was supplemented with 5 mM CaCl_2_ and clotted with 10 nM thrombin in the presence or absence of FXIII for 95 min at 37 °C. Fibrin samples were solubilized by layering plasmin (3.3 μM) on the surface of the clots for 150 min, then further digested with trypsin (2.15 μM) for 90 min at 37 °C. The clear solution obtained was reduced with DTT (20 mM) for 30 min and alkylated with iodoacetamide (25 mM) for 30 min at room temperature. Digestion was stopped by adding 2% formic acid, and the samples were centrifuged to remove all undigested proteins.

Mass spectrometry measurements were performed on a Thermo LTQ XL MS system coupled to an Ultimate 3000 liquid chromatograph. Data were collected over the 350–1400 m/z range, with 3 precursors selected for fragmentation. Peptides were separated on a Waters BEH C18 1x100 mm column using a water/acetonitrile gradient with 0.1% formic acid, as previously described (27). Briefly: the flow rate was set to 0.03 mL/min at 5% acetonitrile for 1 minute, then increased to 44% over 111 minutes. Measurements were repeated using both Collision-Induced Dissociation (CID) and Electron Transfer Dissociation (ETD) fragmentation protocols to optimize sampling of the cross-linked components of the proteins.

**Figure 1:**
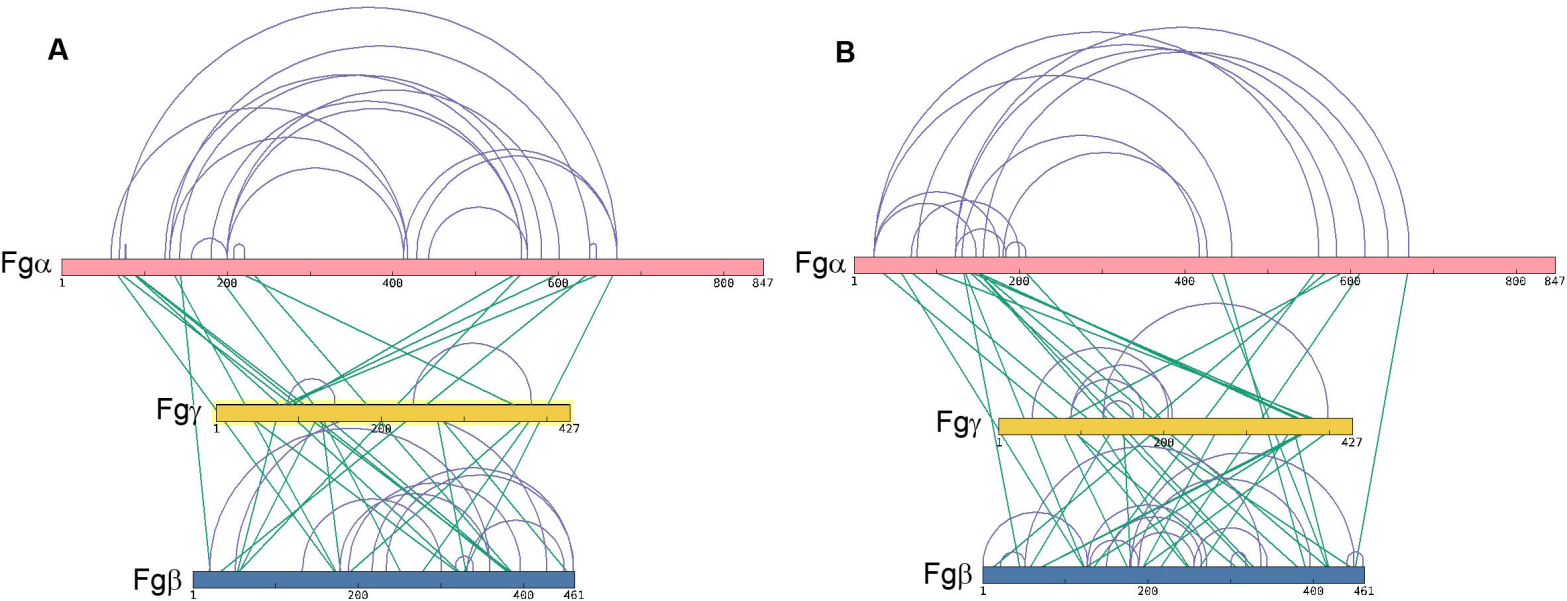
Citrullination increases the number of FXIII cross-linking sites in fibrinogen. Citrullinated and native fibrinogen were supplemented with Ca^2+^ and clotted by thrombin, with or without FXIII. Fibrin clots were sequentially digested with plasmin and trypsin. MS analysis was conducted after separation of the digested peptides by liquid chromatography. Loops display the cross-linking sites between the Gln and Lys side chains identified in the three polypeptide chains (α, red; β, blue; γ, yellow) of the native **(A)** and citrullinated **(B)** fibrinogen molecule. Lilac and green loops indicate cross-links between identical (homo-chain bonds) and different (hetero-chain bonds) polypeptide chains, respectively. The schematic cross-link maps depict a single monomer, though cross-links are actually formed in fibrin polymers, i.e., between different monomers. In the non-citrullinated sample, 27 hetero-links and 29 homo-links were identified **(A)**, while in the citrullinated sample, 36 hetero-links and 30 homo-links were identified **(B)**, with the exact positions reported in the **Supplementary Table 1**.

Data analysis was carried out using the MassAI software package (28). The data were converted to MGF format using MSConvert, and the search space was restricted to the fibrin chains present in the samples. The search settings were as follows: 3 missed cleavages, a maximum peptide mass of 6000 Da, and a minimum length of 5 AA; Carbamidomethyl was set as a fixed modification, and methionine oxidation as a variable modification. The data analysis incorporated citrullination as a variable modification, which facilitated the identification of missed cleavages. This approach enabled the detection of peptides in which trypsin cleavage was compromised due to arginine deimination. For the cross-linking search, isopeptide bond formation between lysine and glutamine was allowed, with a delta mass of -17.027 Da; parent mass tolerance was 200 ppm, and MS2 tolerance was set to 0.5 m/z. The results were filtered to retain only the best-scoring matches. The score limit was set to 10, corresponding to a false discovery rate (FDR) of 1%. **Figure 1**, showing the potential crosslinking sites on native and citrullinated fibrinogen was generated using xiView (29).

### 2.4 Turbidimetric fibrin lysis assay

Turbidimetry was used to compare the lysis kinetics of fibrin clots formed from either citrullinated or native fibrinogen. Fibrinogen at 7.35 μM was supplemented with 5 mM CaCl_2_ and clotted with 10 nM thrombin in a 96-well microtiter plate, in the absence or presence of 20 µg/mL FXIII, for 80 minutes at 37 °C. Clot lysis was initiated by layering plasmin (3.3 μM) on the surface of the clots, and light absorbance was measured at 340 nm over time. Lysis time (LT_10_) for each of at least three replicates in three independent experiments was defined as the time required for the turbidity signal to reach 10% of A_max_ on the descending turbidimetric curve and was expressed in relative units compared with the mean LT_10_ of the non-citrullinated, non-crosslinked sample from the same experiment.

### 2.5 Oscillatory rheometry

The viscoelastic properties of fibrin clots were studied using a cone-and-plate oscillation rheometer, as previously described (30). Citrullinated or native fibrinogen at 7.35 μM was supplemented with 5 mM CaCl_2_, in the absence or presence of 20 μg/mL FXIII. Immediately after the addition of 10 nM thrombin, the clotting mixture was transferred to the stationary plate of a HAAKE RheoStress1 oscillation rheometer (Thermo Scientific, Karlsruhe, Germany). The rheometer’s cone (Titanium, 2° angle, 35 mm diameter) was brought to the gap position, and 2 min after clot initiation, the sample was subjected to an oscillatory shear strain (γ) of 0.015 (corresponding to 1.5% deformation) at 1 Hz. Storage modulus (G’) and loss modulus (G”) were measured continuously for 15 min during clotting in three independent experiments. To determine the gel/fluid transition of the same clots, shear stress (τ) was increased stepwise from 0.01 to 1000 Pa within 5 min, and dynamic viscosity (η) was determined at each step. For numerical description of the flow curves, the maximum bearable strain (γ_max_) at the point of abrupt fall in viscosity to zero (gel/fluid transition) and the critical shear stress (τ_0_) at which viscosity falls to zero were determined.

### 2.6 Kinetic viscoelastometry

Clot firmness and lysis kinetics under dynamic conditions were evaluated using the ClotPro^®^ viscoelastometry device (Enicor GmbH, Munich, Germany), which employs cup-and-pin technology to rapidly assess whole-blood coagulation and lysis. In this system, the pin remains stationary while a rotational force is applied to the cup, and the resulting movement is recorded (31). In our in-house fibrinolytic assay, a mixture of 6.5 μM fibrinogen, 0.17 μM plasminogen, 5 mM CaCl_2_, and 0.19 μM tPA was added to the cup, with or without 20 µg/mL FXIII, and clotted with 97 nM thrombin in a total volume of 340 µL. To meet the substantial material requirements of this assay, the fibrinogen concentration used in this measurement was slightly different from the 7.35 µM concentration applied in the turbidimetric and rheometric measurements. However, Risman et al. have demonstrated that concentrations varying in this range result in only negligible changes to the fibrin structure (32). Maximal clot firmness (MCF, expressed as viscoelastometric amplitude that characterises the clotting-mediated reduction in cup rotation: 50 mm refers to a 50% decline in the stretching of the clot) and lysis time (the time from reaching a viscoelastic amplitude of 2 mm [clotting time] to a 50% decrease in clot firmness after the maximum) were determined in three independent experiments in the ClotPro^®^ system and reported in relative units compared with the non-citrullinated, non-crosslinked sample from the same experiment.

### 2.7 Statistical analysis

Differences of data distributions compared to control groups in the turbidimetric, viscoelastometric, and rheometric measurements were statistically evaluated using a bootstrap variant of the Mann-Whitney U test (Statistics and Machine Learning Toolbox v.12.5 of MatlabR2023a) as previously described (33). The detailed description of the statistical framework and pairwise comparisons, including bootstrap-derived test statistic *U*, *P*-values, effect sizes, and test power, are presented in **Supplementary Material**.

## 3 Results

### 3.1 Citrullination increases the number of potential FXIII cross-linking sites on fibrinogen

Citrullination of fibrinogen was confirmed by MS analysis, which also showed that the number of citrullinated fragments was proportional to the concentration of the PAD4 enzyme used (**Supplementary Table 1**). Further evaluation revealed a moderate increase in the number of potential cross-linking sites following prior enzymatic citrullination of fibrinogen. In the non-citrullinated sample, 29 high-confidence isopeptide bonds were detected between identical fibrinogen chains (homo-chain bonds, e.g. γ-γ) and 27 between different chains (hetero-chain bonds, e.g. α-γ; **Figure 1A**), whereas the citrullinated sample exhibited 30 and 36, respectively (**Figure 1B**; for exact positions of crosslinks in citrullinated and non-citrullinated fibrin, see **Supplementary Table 2**). This corresponds to an 18% increase in the total number of potential cross-linking sites due to citrullination. Our conclusions are based on relative detection patterns and reproducible peptide evidence rather than absolute peptide counts, minimizing the impact of potential digestion efficiency differences caused by citrullination.

### 3.2 Citrullination modulates the FXIII-induced lytic resistance of fibrin clots

The lysis of fibrin clots formed from partially citrullinated fibrinogen was studied in two experimental setups: in the two-phase turbidimetric assay, plasmin was applied to the surface of pre-formed fibrin clots, whereas in the intrinsic viscoelastometric ClotPro^®^ assay, the fibrinolytic enzymes were homogenously dispersed within the forming clots.

**Figure 2:**
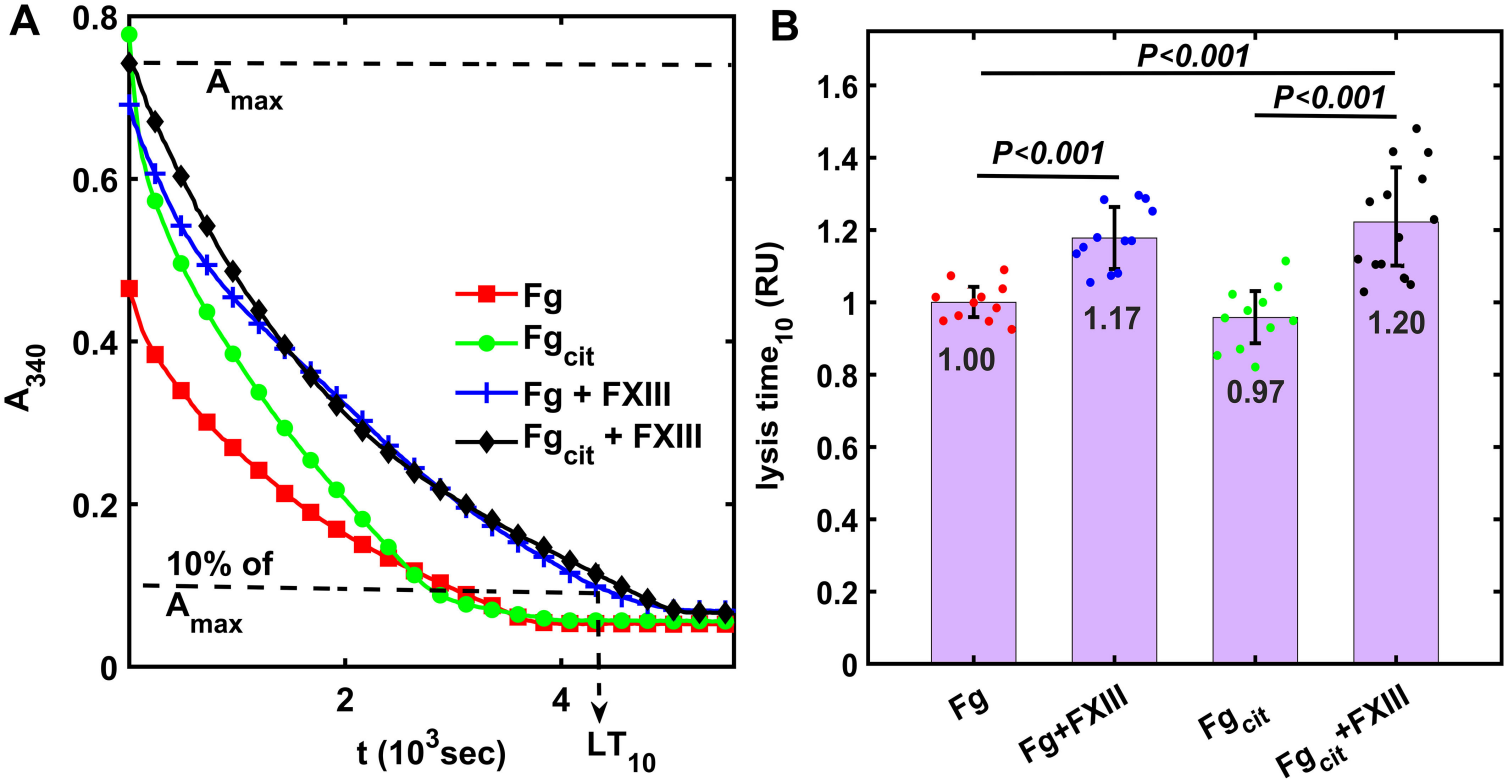
FXIII exerts a stronger stabilizing effect in the extrinsic lysis of citrullinated than in non-citrullinated fibrin. **(A)** Native (Fg) or citrullinated fibrinogen (Fg_cit_) was supplemented with Ca^2+^ and clotted with thrombin in the presence or absence of FXIIIa. After clotting, lysis was initiated by adding plasmin to the clots, and it was monitored by measuring absorbance at 340 nm (A_340_). Representative curves show the mean absorbance of 3 replicates. Lysis times were defined as the time required to reduce the absorbance to 10% of the maximal value (LT_10_), illustrated with the dashed lines. **(B)** Relative LT_10_ compared with the median lysis time of the non-citrullinated, non-crosslinked sample from the same experiment. Bars and error bars represent the median and interquartile range; dots show individual replicates (n=3). P-values were determined according to a bootstrap variant of the Mann-Whitney U test as illustrated in **Supplementary Figure 2**.

**Figure 3:**
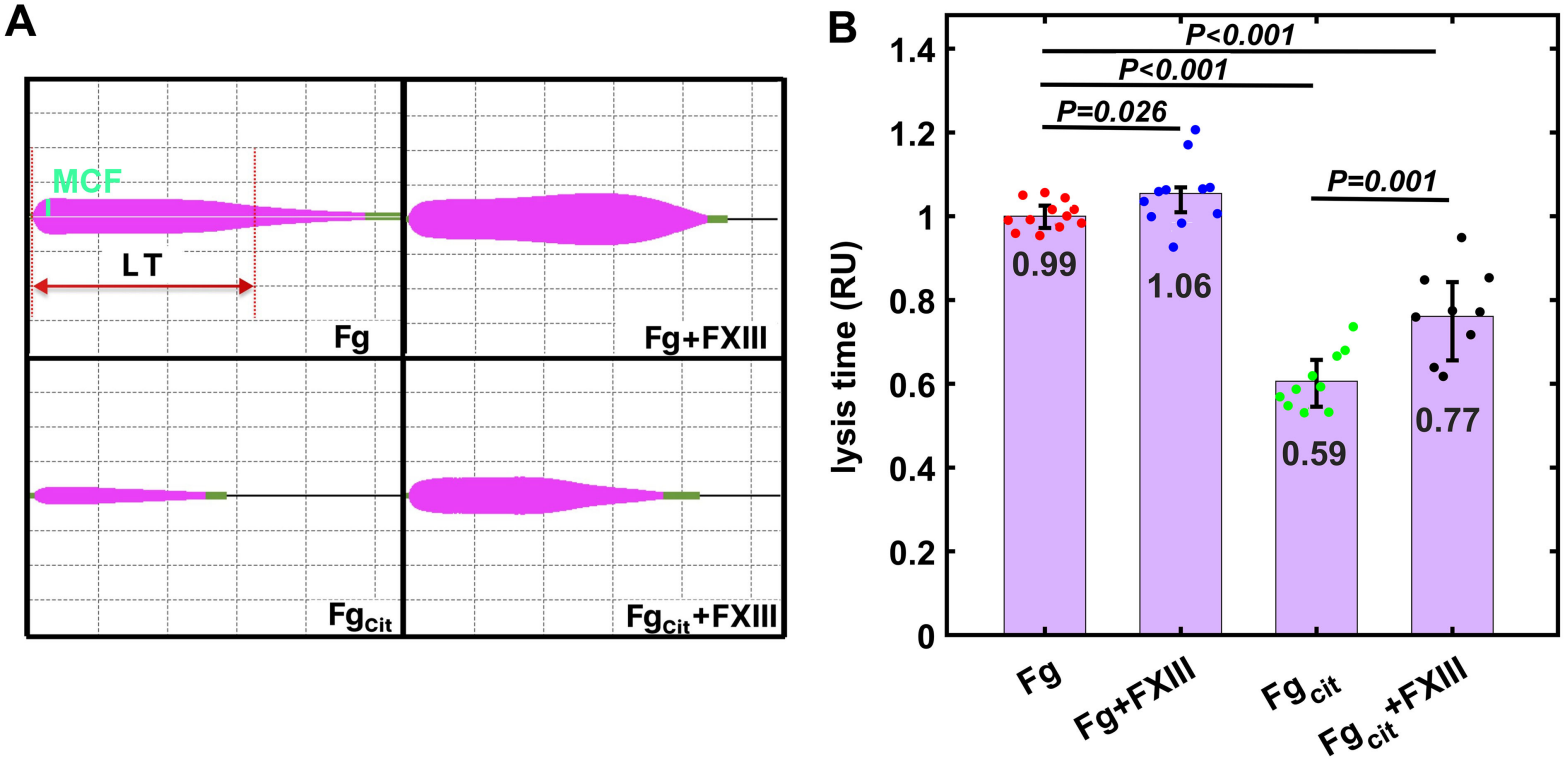
FXIII exerts a stronger stabilizing effect in the intrinsic lysis of citrullinated than in non-citrullinated fibrin. **(A)** For ClotPro^®^ measurements, native (Fg) or citrullinated fibrinogen (Fg_cit_) was supplemented with Ca^2+^, plasminogen and tPA, then clotted with thrombin in the presence or absence of FXIIIa. Representative thromboelastograms for the indicated experimental conditions are shown, with one grid square corresponding to 10 mm vertically (clot firmness) and 10 minutes horizontally (time). Lysis times (LT) were defined as the duration from the onset of clot formation to the point at which the maximal clot firmness (MCF) is reduced by 50%, as illustrated in the top-left panel. **(B)** Relative LT compared with the median lysis time of the non-citrullinated, non-crosslinked sample from the same experiment. Bars and error bars represent the median and interquartile range; dots show individual replicates (n=3). P-values were determined according to a bootstrap variant of the Mann-Whitney U test as described in **Supplementary Figure 3**.

FXIII-mediated crosslinking prolonged the lysis time of native fibrin clots in both experimental settings, with an 17% increase in the two-phase assay (**Figure 2B**) and a 7% increase in the intrinsic assay (**Figure 3B**). FXIII-mediated crosslinking increased the clot lysis time of citrullinated fibrin by 24% in the two-phase assay (**Figure 2B**) and by 31% in the homogeneous intrinsic assay (**Figure 3B**). Notably, citrullination on its own significantly affected lysis time only in the intrinsic lysis assay, reducing it by 40% (**Figure 3B**). FXIII crosslinking partially reversed this effect, and the relative increment in lysis time attributable to FXIIIa was larger in citrullinated fibrin (31%) than in native fibrin (7%) (**Figure 3B**). A similar trend in the impact of FXIIIa on the lytic susceptibility of citrullinated fibrin was observed in the two-phase lysis assay: 24% prolongation of the lysis time of citrullinated fibrin after crosslinking vs. 7% in non-citrullinated fibrin (**Figure 2B**). Regarding the combined effect of FXIII crosslinking and citrullination on the lytic susceptibility of fibrin, the two lysis assays showed discordant trends: a 20% prolongation of the lysis time of crosslinked citrullinated fibrin compared to non-crosslinked non-citrullinated fibrin in the two-phase assay (**Figure 2B**) and a 22% shortening in the homogeneous ClotPro^®^ assay (**Figure 3B**). Because the output signal of the ClotPro^®^ depends not only on the degree of lysis but also on the biomechanical strength of the fibrin clots, additional direct measurements of the viscoelastic properties of fibrin were performed to clarify the basis of this discrepancy.

### 3.3 Citrullination reduces mechanical clot stability

**Figure 4:**
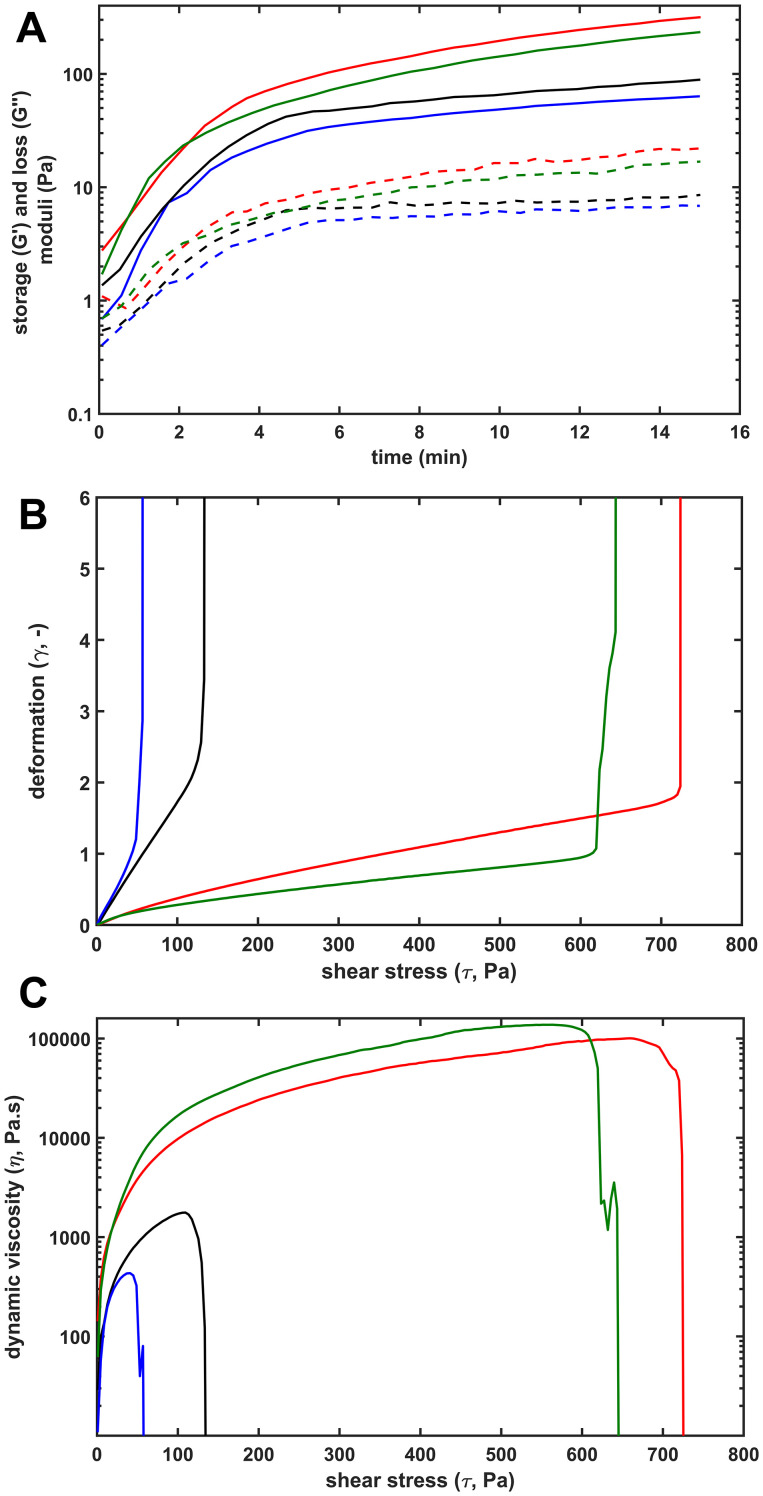
Citrullination decreases, whereas FXIII increases the mechanical stability of fibrin as assessed by rheometry. **(A)** Representative curves show the development of clot strength (G’ storage modulus: solid line, G” loss modulus: dashed line) during thrombin-induced fibrin formation in the absence of FXIII from native (black) or citrullinated (blue) fibrinogen, or in the presence of FXIII from native (red) or citrullinated (green) fibrinogen. **(B, C)** After 15 min of clotting, increasing shear stress (τ) was applied to the same clot in the measurement gap, and the resulting deformation (γ) was measured. The representative curves depict the deformation, γ, **(B)** and the computed dynamic viscosity, η, **(C)**. Numerical data with statistical analysis are summarized in **Table 1**.

Rheological measurements showed that fibrin formed from citrullinated fibrinogen is mechanically less stable, as evidenced by 28% and 26% reductions in storage (G′, from 90.6 to 65.2 Pa) and loss (G″, from 9.65 to 7.16 Pa) moduli, respectively (**Figure 4A**; **Table 1**). Additionally, the 15% increase in loss tangent (G″/G′, from 0.098 to 0.113) upon citrullination suggests an overall decline in elasticity, consistent with the observed decrease in γ_max_ (**Figure 4C**; **Table 1**). Compared with the native control, the flow curves of citrullinated clots exhibited significantly lower dynamic viscosity, and the gel-to-fluid transition, marking clot disassembly, occurred at a critical shear stress 24% lower (125.2 Pa and 94.9 Pa in native and citrullinated clots, respectively), indicating reduced resistance to shear stress upon citrullination (**Figure 4B**; **Table 1**).

**Table 1:** FXIII exerts a stronger mechanical stabilizing effect in citrullinated than in non-citrullinated fibrin as assessed by rheology.

Clot type	G’ (Pa)	G” (Pa)	G”/G’ (-)	γ_max_ (-)	τ_0_ (Pa)
Fg	90.6 [74.1-123.0]	9.65 [6.04-12.31]	0.098 [0.092-0.107]	3.44 [1.89-3.78]	125.2 [93.1-145.6]
Fg + FXIIIa	273.9^#^ [267.2-316.4]	19.46^#^ [19.12-20.36]	0.068^#^ [0.063-0.074]	1.79^#^ [1.32-2.01]	729.3^#^ [695.7-812.1]
relative FXIIIa effect	3.02	2.02	0.70	0.52	5.82
Fg_cit_	65.2* [43.5-70.6]	7.16* [5.01-7.92]	0.113* [0.109-0.119]	2.93 [1.76-3.59]	94.9* [56.9-117.4]
Fg_cit_ + FXIIIa	209.3*^#^ [160.8-263.9]	14.20*^#^ [12.65-17.65]	0.068^#^ [0.065-0.078]	3.66* [2.29-4.60]	586.9^#^ [447.4-849.8]
relative FXIIIa effect	3.21	1.98	0.60	1.25	6.18

Native (Fg) or citrullinated fibrinogen (Fg_cit_) was supplemented with CaCl_2_ and clotted with thrombin, with or without FXIII, within the rheometer measurement gap. The forming clot was subjected to an oscillatory shear strain, and G’, G’’ and the loss tangent (G”/G’) were determined. In a second phase, stepwise increasing shear stress was applied to the clot, and the maximal bearable strain (γ_max_) and the critical shear stress (τ_0_) were determined. Median values [bottom – top quartiles] are shown. Asterisks (*) indicate significant differences due to citrullination, while hashtags (#) indicate significant differences caused by FXIII-mediated crosslinking according to the Mann-Whitney U test (n=3 with 5 replicates in each independent group), as presented in **Supplementary Figures 5–7**.

### 3.4 Citrullination modulates the FXIII-mediated mechanical stabilization of fibrin clots

**Figure 5:**
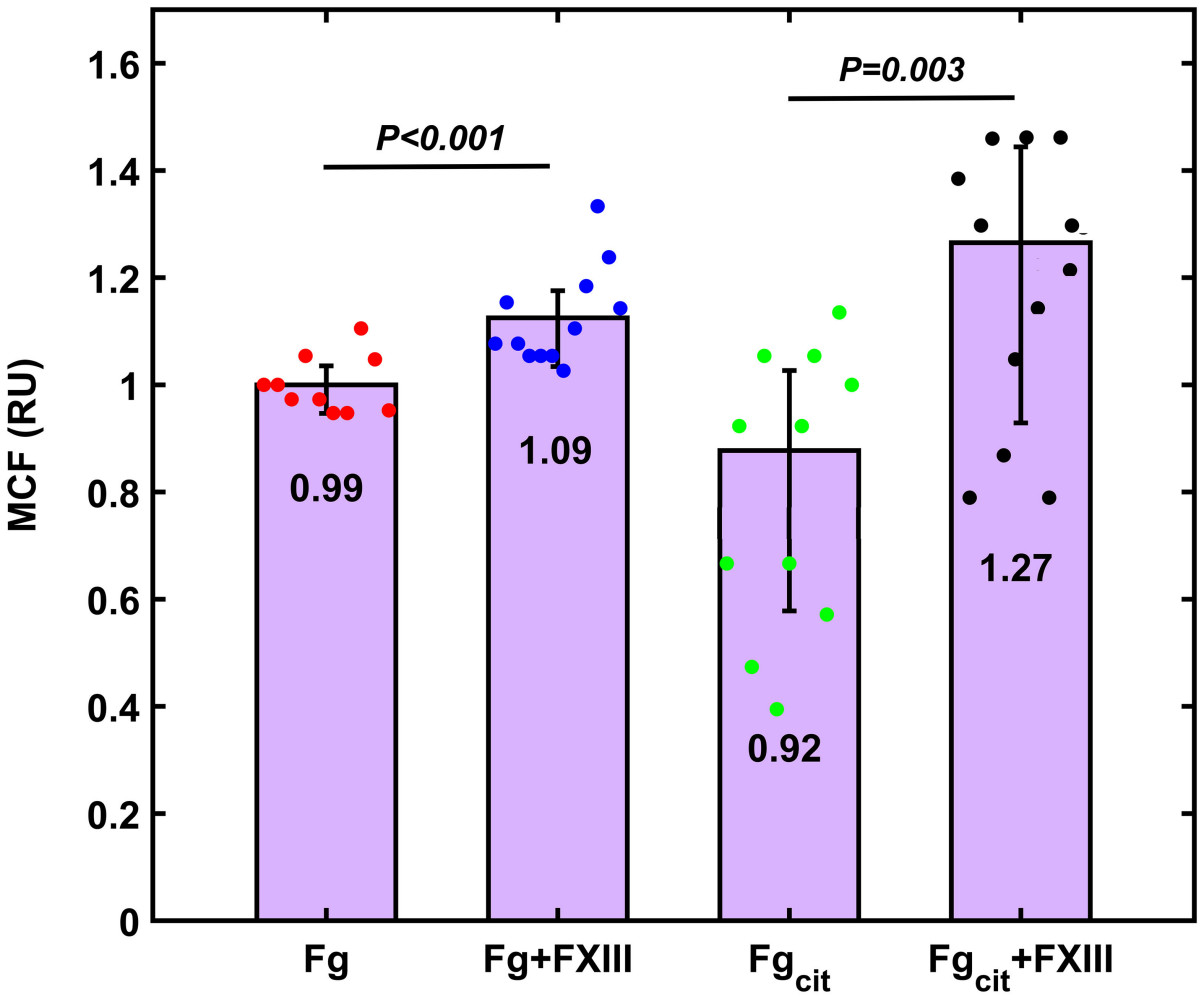
FXIII exerts a stronger mechanical stabilizing effect in citrullinated than in non-citrullinated fibrin as assessed by viscoelastometry. Mixtures of native (Fg) or citrullinated fibrinogen (Fg_cit_), plasminogen, CaCl_2_, and tPA +/- FXIII were clotted with thrombin in the rotating cup of the ClotPro^®^ device and clot firmness was monitored as illustrated in **Figure 3**. Maximal clot firmness (MCF) is shown in relative units (RU) compared with the median MCF of the non-citrullinated, non-crosslinked sample from the same experiment. Bars and error bars represent the median and interquartile range; dots show individual replicates (n=3). P-values were determined according to a bootstrap variant of the Mann-Whitney U test as described in **Supplementary Figure 4**.

Oscillation rheometry demonstrated that FXIII-mediated covalent crosslinking markedly enhanced the mechanical stability of both native and citrullinated fibrin compared with their non-crosslinked counterparts. For the non-citrullinated case, the storage modulus (G’), loss modulus (G”), and the critical shear stress required for the gel/fluid transition (τ_0_) increased by 3.0, 2.0, and 5.8 times, respectively, and the loss tangent (G”/G’) decreased by 31% due to crosslink formation, suggesting that energy loss during deformation is lower and the polymer undergoes less irreversible deformation (**Table 1**). In the citrullinated case, the increment factors were 3.2, 2.0, and 6.2, respectively, while the loss tangent decreased by 40%. Despite preliminary citrullination leading to crosslink-derived stabilization to a relatively higher degree, the G’, G”, G”/G’, and τ_0_ values of crosslinked fibrin indicated a general decline in stability associated with citrullination (Fg + FXIII vs. Fg_cit_+FXIII in **Table 1**). The maximal bearable strain (γ_max_) decreased in native fibrin as a result of crosslink formation (from 3.4 to 1.8) and increased in the crosslinked sample upon citrullination (from 1.8 to 3.7, **Figure 4B**; **Table 1**). The MCF in the viscoelastometric ClotPro^®^ assay increased by 10% after crosslinking of native fibrin by FXIIIa, whereas this increase was 38% in citrullinated fibrin (**Figure 5**). Overall, the changes observed in the MCF indicated that the formation of crosslinks resulted in a greater relative stabilization of citrullinated fibrin. However, the process of citrullination did not affect the overall stability related to crosslinks (as seen in the comparison of Fg + FXIII and Fgcit + FXIII in **Figure 5**) nor did it lead to a decrease in stability on its own.

## 4 Discussion

In immunothrombosis, neutrophil extracellular traps (NETs) form alongside fibrin deposition (34, 35). In this process, peptidylarginine deiminases (PAD), particularly PAD2 and PAD4, either become exposed on the neutrophil cell surface or are released into the surrounding microenvironment. This activity catalyzes the citrullination of extracellular proteins, as evidenced by the identification of citrullinated fibrin(ogen) in venous thrombi (15). Furthermore, citrullinated fibrin(ogen) has also been detected in the plasma clots of patients with rheumatoid arthritis, as well as in cases of bacteremia and cancer (21, 36, 37). These conditions are well-known to be associated with an increased risk of thrombosis (38–40), highlighting the importance of considering clot stability in relation to citrullinated fibrin and its crosslinking. Citrullination alters the electrostatic properties of the fibrinogen molecule by converting positively charged arginine residues into neutral citrullines. This modification changes the polymerization pattern of fibrin monomers, resulting in a low-porosity meshwork of thinner, densely packed fibers (16). Additionally, citrullination disrupts critical thrombin cleavage sites in fibrinogen, particularly hindering the release of fibrinopeptides A and B. This disruption slows down the generation and polymerization of fibrin monomers, adding a kinetic aspect that modifies the structure of fibrin (20, 21, 26). Given these citrullination-related structural changes, the spatial distribution of glutamine and lysine residues—substrates for FXIIIa—may also be affected. This could lead to variations in the number of crosslinks between fibrin monomers and the functional characteristics of the fibrin matrix. Our current mass spectrometry analysis has revealed that citrullination increases the number of sites in fibrin where FXIIIa facilitates crosslinking. The identified cross-linking sites, illustrated in **Figure 1**, include the well-characterized α–α, α–γ, and γ–γ chain linkages (41), along with the recently reported α–β, β–β, and β–γ cross-links (42, 43).

In line with our previous study (15), citrullination weakened non-crosslinked fibrin clots as assessed by rheology. The values of the storage and loss moduli and critical shear stress indicated that citrullinated fibrin was more easily deformable and mechanically less stable than its native counterpart. FXIII-mediated crosslinking mechanically stabilized both citrullinated and non-citrullinated fibrin. However, the relative increases in viscoelastic parameters – storage modulus, critical shear stress, and maximal clot firmness – and the decrease in loss tangent were greater in citrullinated fibrin (**Figures 4**, **5**; **Table 1**). The increased number of crosslinking sites provides a mechanistic link to the improved mechanical stabilizing effect of FXIIIa in citrullinated fibrin.

The modest degree of fibrinogen citrullination used in the current study did not affect the lysis kinetics of fibrin in the two-phase turbidimetric assay (**Figure 2B**), – in contrast to the lysis inhibition previously observed by us in non-crosslinked fibrin formed from more heavily citrullinated fibrinogen (15). In addition, when the lytic process was monitored using a signal that reflects not only the degree of lysis but also clot firmness (shear resistance), as in our viscoelastometric ClotPro^®^ assay, citrullination resulted in faster resolution of non-crosslinked fibrin with enzymes homogenously dispersed in the clot (**Figure 3B**). This outcome likely reflects the mechanical weakening effect of citrullination observed previously [] and confirmed under the conditions of the current study, as discussed above. FXIIIa slightly prolonged the lysis time of non-citrullinated fibrin in both the extrinsic two-phase and the intrinsic homogeneous lysis assays. A much more pronounced increase in lytic resistance attributable to FXIII crosslinking was observed in citrullinated fibrin. The enhanced anti-fibrinolytic effect of FXIIIa in citrullinated fibrin can also be attributed to the increased number of crosslinks formed in this fibrin meshwork.

It is noteworthy that, in both intrinsic and extrinsic lysis setups, the relative increase in lysis times due to crosslinking was more pronounced when fibrinogen was citrullinated. In addition to lysines, arginines serve as plasmin cleavage sites during fibrinolysis. The question arises whether the more pronounced increase in lysis times of FXIII-crosslinked samples in the citrullinated case is the consequence of a diminished number of potential plasmin cleavage sites. However, this suggestion can be rejected because, in the absence of FXIIIa, citrullination alone did not prolong lysis time.

Fibrin is recognized for its significant role in enhancing thrombin-mediated activation of FXIII, and modifications to the structure of fibrin may influence this effect (44). It is important to note, however, that such an enhancing effect requires the presence of the B subunit (45, 46). Given that recombinant human FXIII-A_2_ was employed in this study, any observed differences in FXIII activation between native and citrullinated fibrinogen are unlikely to be due to fibrin-mediated enhancement of activation. Nonetheless, this mechanism may be pertinent in the context of plasma FXIII; therefore, further investigation is warranted to elucidate this possibility.

In line with previous findings, the maximal bearable strain (γ_max_) decreased after factor XIII-mediated crosslinking, attributable to reduced extensibility resulting from inhibition of protofibril sliding (47, 48). Notably, the γ_max_ of citrullinated, crosslinked fibrin exceeded that of non-citrullinated, crosslinked fibrin. The increased maximal bearable strain may arise from altered crosslink positions in the citrullinated fibrin, as revealed by MS (**Supplementary Table 1**).

It is important to note that α_2_-antiplasmin was not included in the purified experimental system used in our current study. However, given the established role of FXIII in crosslinking α_2_-antiplasmin to enhance fibrin stability and contribute to fibrinolytic resistance, it is essential to investigate the involvement of α_2_-antiplasmin in this mechanism. Therefore, further research into whether FXIII-mediated incorporation of α_2_-antiplasmin into fibrin is affected by citrullinated fibrinogen would be very informative. Additionally, exploring how these modifications may influence the susceptibility of the resulting fibrin network to fibrinolysis would also be valuable.

*In vivo*, partial citrullination of fibrinogen may occur, leading to the formation of fibrin clots that contain fewer fibrin monomers than those formed from native fibrinogen. While the molecular changes caused by citrullination could theoretically promote crosslinking by reducing electrostatic repulsion, the overall stabilizing effect is not achieved. This is because the reduced availability of fibrin monomers limits the extent of clot formation and crosslinking. Additionally, in rheumatoid arthritis, autoantibodies against citrullinated FXIIIa have been identified and are associated with decreased transglutaminase activity (49). However, direct evidence of citrullination of FXIII itself and of its impact on activity remains limited. *In vivo*, both fibrinogen and FXIII may undergo citrullination simultaneously. Therefore, the combined effects of reduced fibrin monomer availability, decreased FXIII activity, and the potentially enhanced crosslinking of citrullinated fibrin merit further investigation.

In conclusion, our *in vitro* studies identified a new mechanism that reinforces the prothrombotic effects of NETs. Although the presence of citrullinated monomers in fibrin compromises its biomechanical stability, citrullination promotes spatial rearrangements in the fibrin matrix that allow additional crosslinks to form via FXIIIa, which efficiently counteract mechanical and lytic destabilization. These *in vitro* findings indicate trends whose relative significance for the *in vivo* stability of thrombi depends on the degree of citrullination. This newly described FXIII-dependent mechanism requires NETs, which have been observed in pathological venous and arterial thrombi but not in physiological haemostatic blood clots. This NET-related discrimination between pathological thrombi and clots that prevent bleeding explains the favorable safety profile of a new class of anticoagulants that target FXIII (50).

## Data Availability

All raw data analysed in this publication are deposited in the Zenodo public repository under DOI 10.5281/zenodo.21411915.
