# Supplementary material for: Increased number of crosslinking sites for factor XIII counteracts the mechanical and lytic destabilization of fibrin caused by citrullination of fibrinogen
Source: Front Immunol. 2026 Jul 27;17:1897298. doi: 10.3389/fimmu.2026.1897298 (PMC13453679; doi:10.3389/fimmu.2026.1897298)
Supplement: Supplementary file 1 [file DataSheet1.docx]

**Increased Number of Crosslinking Sites for Factor XIII Counteracts the Mechanical and Lytic Destabilization of Fibrin Caused by Citrullination of Fibrinogen**

Kornélia Guzmits, Erzsébet Komorowicz, Olivér Ozohanics, Krasimir Kolev, Anna Tanka-Salamon

**Supplementary data**

**Figure S1: Clottability of fibrinogen citrullinated to varying extents.** Thrombin was added to native fibrinogen (red) or to fibrinogen citrullinated with 270 (green), 620 (blue), or 800 (black) ng/mL PAD4. Fibrin mesh formation was monitored by turbidimetry at 340 nm. Representative curves show changes in absorbance (A_340_) over time.

**Table S1: Relative amounts of citrulline-bearing degradation fragments of fibrinogen citrullinated to various extents.** Fibrinogen (9.1 μM) was treated with PAD4 at 270, 620 or 800 ng/mL for 1.5 hours at 37 °C in the presence of CaCl_2_ (6.8 mM) and DTT (0.68 **m**M). The degree of citrullination was investigated by MS. Numbers indicate peak areas of randomly selected peptides bearing citrulline at the positions shown.

|  | | **grade of citrullination** | | |
| --- | --- | --- | --- | --- |
| **Fg chain** | **position** | **low** | **medium** | **high** |
| α | 414 | 1.623.252 | 1.700.659 | 4.102.020 |
| α | 262 | 404.162 | 440.672 | 800.525 |
| α | 361 | 1.298.015 | 1.543.298 | 2.719.749 |
| α | 335 | 10.640.691 | 8.553.936 | 12.162.043 |
| α | 510 | 51.636 | 105.620 | 418.493 |
| α | 541 | 1.800.409 | 2.564.918 | 3.691.125 |
| β | 50 | 3.786.171 | 5.537.616 | 10.624.847 |

**Table S2 Positions of crosslinks formed by FXIIIa in fibrinogen.** Native (A) and citrullinated (B) fibrinogen (9.1 μM) was supplemented with CaCl_2_ (5 mM) and clotted with thrombin (10 nM) +/- FXIII (20 μg/mL). Cross-linked fibrin samples were solubilized with plasmin (3.3 μM), then further digested with trypsin (2.15 μM). Crosslink formation was investigated by MS. Numbers indicate positions according to the UniProt database.

| **A** | **Fg chain I.** | **position** | **amino acid** | **Fg chain II.** | **position** | **amino acid** |
| --- | --- | --- | --- | --- | --- | --- |
| 1 | α | 70 | Lys | α | 671 | Gln |
| 2 | α | 78 | Lys | α | 77 | Gln |
| 3 | α | 125 | Lys | α | 563 | Gln |
| 4 | α | 157 | Lys | α | 200 | Gln |
| 5 | α | 208 | Lys | α | 221 | Gln |
| 6 | α | 413 | Lys | α | 200 | Gln |
| 7 | α | 413 | Lys | α | 671 | Gln |
| 8 | α | 413 | Lys | α | 671 | Gln |
| 9 | α | 418 | Lys | α | 60 | Gln |
| 10 | α | 418 | Lys | α | 131 | Gln |
| 11 | α | 429 | Lys | α | 671 | Gln |
| 12 | α | 444 | Lys | α | 563 | Gln |
| 13 | α | 556 | Lys | α | 200 | Gln |
| 14 | α | 556 | Lys | α | 181 | Gln |
| 15 | α | 580 | Lys | α | 143 | Gln |
| 16 | α | 601 | Lys | α | 200 | Gln |
| 17 | α | 638 | Lys | α | 646 | Gln |
| 18 | α | 638 | Lys | α | 131 | Gln |
| 19 | β | 21 | Lys | β | 301 | Gln |
| 20 | β | 133 | Lys | β | 339 | Gln |
| 21 | β | 178 | Lys | β | 460 | Gln |
| 22 | β | 217 | Lys | β | 52 | Gln |
| 23 | β | 234 | Lys | β | 359 | Gln |
| 24 | β | 318 | Lys | β | 359 | Gln |
| 25 | β | 396 | Lys | β | 189 | Gln |
| 26 | β | 428 | Lys | β | 332 | Gln |
| 27 | β | 449 | Lys | β | 460 | Gln |
| 28 | γ | 88 | Lys | γ | 144 | Gln |
| 29 | γ | 381 | Lys | γ | 239 | Gln |
| 30 | α | 60 | Gln | β | 393 | Gln |
| 31 | α | 60 | Gln | β | 47 | Lys |
| 32 | α | 77 | Gln | β | 22 | Lys |
| 33 | α | 131 | Gln | β | 323 | Lys |
| 34 | α | 143 | Gln | β | 22 | Lys |
| 35 | α | 224 | Lys | β | 178 | Lys |
| 36 | α | 563 | Gln | β | 392 | Lys |
| 37 | α | 646 | Gln | β | 396 | Lys |
| 38 | α | 671 | Gln | β | 178 | Lys |
| 39 | α | 77 | Gln | γ | 58 | Lys |
| 40 | α | 77 | Gln | γ | 120 | Lys |
| 41 | α | 181 | Gln | γ | 75 | Lys |
| 42 | α | 200 | Gln | γ | 62 | Lys |
| 43 | α | 563 | Gln | γ | 88 | Lys |
| 44 | α | 618 | Gln | γ | 380 | LYs |
| 45 | α | 671 | Gln | γ | 125 | Lys |
| 46 | β | 52 | Gln | γ | 266 | Lys |
| 47 | β | 52 | Gln | γ | 266 | LYs |
| 48 | β | 180 | Gln | γ | 127 | Lys |
| 49 | β | 180 | Gln | γ | 75 | Lys |
| 50 | β | 256 | Gln | γ | 35 | Lys |
| 51 | β | 271 | Gln | γ | 88 | Lys |
| 52 | β | 332 | Gln | γ | 125 | Lys |
| 53 | β | 332 | Gln | γ | 125 | Lys |
| 54 | β | 332 | Gln | γ | 380 | Lys |
| 55 | β | 393 | Gln | γ | 385 | Lys |
| 56 | β | 460 | Gln | γ | 127 | Lys |

| **B** | **Fg chain I.** | **position** | **amino acid** | **Fg chain II.** | **position** | **amino acid** |
| --- | --- | --- | --- | --- | --- | --- |
| 1 | α | 70 | Lys | α | 200 | Gln |
| 2 | α | 123 | Lys | α | 184 | Gln |
| 3 | α | 123 | Lys | α | 671 | Gln |
| 4 | α | 123 | Lys | α | 618 | Gln |
| 5 | α | 148 | Lys | α | 25 | Gln |
| 6 | α | 157 | Lys | α | 646 | Gln |
| 7 | α | 176 | Lys | α | 25 | Gln |
| 8 | α | 208 | Lys | α | 184 | Gln |
| 9 | α | 418 | Lys | α | 131 | Gln |
| 10 | α | 427 | Lys | α | 181 | Gln |
| 11 | α | 457 | Lys | α | 25 | Gln |
| 12 | α | 562 | Lys | α | 25 | Gln |
| 13 | α | 583 | Lys | α | 77 | Gln |
| 14 | β | 127 | Lys | β | 271 | Gln |
| 15 | β | 127 | Lys | β | 1 | Gln |
| 16 | β | 130 | Lys | β | 189 | Gln |
| 17 | β | 178 | Lys | β | 256 | Gln |
| 18 | β | 265 | Lys | β | 150 | Gln |
| 19 | β | 318 | Lys | β | 301 | Gln |
| 20 | β | 337 | Lys | β | 52 | Gln |
| 21 | β | 344 | Lys | β | 256 | Gln |
| 22 | β | 396 | Lys | β | 189 | Gln |
| 23 | β | 441 | LYs | β | 460 | Gln |
| 24 | β | 449 | Lys | β | 180 | Gln |
| 25 | γ | 88 | Lys | γ | 210 | Gln |
| 26 | γ | 88 | Lys | γ | 176 | Gln |
| 27 | γ | 127 | Lys | γ | 398 | Gln |
| 28 | γ | 127 | Lys | γ | 163 | Gln |
| 29 | γ | 205 | Lys | γ | 41 | Gln |
| 30 | α | 25 | Gln | β | 318 | Lys |
| 31 | α | 52 | Lys | β | 129 | Gln |
| 32 | α | 131 | Gln | β | 392 | Lys |
| 33 | α | 131 | Gln | β | 47 | Lys |
| 34 | α | 131 | Gln | β | 127 | Lys |
| 35 | α | 143 | Gln | β | 428 | Lys |
| 36 | α | 143 | Gln | β | 428 | Lys |
| 37 | α | 143 | Gln | β | 337 | Lys |
| 38 | α | 429 | Lys | β | 421 | Gln |
| 39 | α | 444 | Lys | β | 393 | Gln |
| 40 | α | 563 | Gln | β | 127 | Lys |
| 41 | α | 618 | Gln | β | 209 | Lys |
| 42 | α | 671 | Gln | β | 449 | Lys |
| 43 | α | 60 | Gln | γ | 120 | Lys |
| 44 | α | 77 | Gln | γ | 406 | Lys |
| 45 | α | 123 | Lys | γ | 398 | Gln |
| 46 | α | 131 | Gln | γ | 381 | Lys |
| 47 | α | 131 | Gln | γ | 381 | Lys |
| 48 | α | 131 | Gln | γ | 385 | Lys |
| 49 | α | 143 | Gln | γ | 140 | Lys |
| 50 | α | 200 | Gln | γ | 205 | Lys |
| 51 | α | 580 | Lys | γ | 176 | Gln |
| 52 | α | 606 | Lys | γ | 65 | Gln |
| 53 | β | 1 | Gln | γ | 196 | Lys |
| 54 | β | 52 | Gln | γ | 140 | Lys |
| 55 | β | 52 | Gln | γ | 381 | Lys |
| 56 | β | 52 | Gln | γ | 385 | Lys |
| 57 | β | 109 | Gln | γ | 380 | Lys |
| 58 | β | 180 | Gln | γ | 151 | Lys |
| 59 | β | 180 | Gln | γ | 75 | Lys |
| 60 | β | 189 | Gln | γ | 266 | Lys |
| 61 | β | 256 | Gln | γ | 88 | Lys |
| 62 | β | 256 | Gln | γ | 120 | Lys |
| 63 | β | 256 | Gln | γ | 356 | Lys |
| 64 | β | 332 | Gln | γ | 140 | Lys |
| 65 | β | 421 | Gln | γ | 356 | Lys |
| 66 | β | 460 | Gln | γ | 170 | Lys |

**Mann-Whitney U test for differences between the compared groups**

The test statistic *U* was calculated using a Bootstrap procedure (with 10,000 pseudo-realisations) that constructs the conditional distributions of *U* under the null hypothesis (identical parameter distributions) and the alternative hypothesis (different parameter distributions) [Balog et al]. The medians [bottom-top quartiles] of the distributions being compared are indicated in the figure legends. The figures show the cumulative density functions (*CDF*s) of the two *U* statistics, the estimated probability of rejecting a true null hypothesis (*p*-value), the common language effect size (*f*), which indicates the probability that a random value from Population 1 will be larger than a random value from Population 2, the observed *U* value (*U_obs_*), the critical *U* value (*U_crt_*), and the power (1−*β*) of the test at a significance level of *α*=0.05 for the indicated pairs of groups.

*Balog Virág K, Csikós P, Raska A, Baráth B, Molnár K, Nikolova N, Tenekedjiev K, Kolev K, Wohner N. Cell-dependent antithrombotic effect of tranexamic acid. Front Immunol 2026; 17:1820813. doi: 10.3389/fimmu.2026.1820813*

**Figure S2. Comparison of lysis times of different fibrin clots measured by turbidimetry as described in Fig. 2.** A: Native non-crosslinked fibrin (0.9992 [0.9524-1.032] RU) vs. native crosslinked fibrin (1.17 [1.107-1.268] RU). B: Native non-crosslinked fibrin (0.9992 [0.9524-1.032] RU) vs. citrullinated non-crosslinked fibrin (0.9569 [0.8856-1.017] RU). C: Native non-crosslinked fibrin (0.9992 [0.9524-1.032] RU) vs. citrullinated crosslinked fibrin (1.204 [1.105-1.341] RU). D: Citrullinated non-crosslinked fibrin (0.9569 [0.8856-1.017] RU) vs. citrullinated crosslinked fibrin (1.204 [1.105-1.341] RU).

**Figure S3. Comparison of lysis times of different fibrin clots measured by viscoelastometry as described in Fig. 3.** A: Native non-crosslinked fibrin (0.9918 [0.9767-1.023] RU) vs. native crosslinked fibrin (1.059 [1.001-1.068] RU). B: Native non-crosslinked fibrin (0.9918 [0.9767-1.023] RU) vs. citrullinated non-crosslinked fibrin (0.59 [0.5476-0.6662] RU). C: Native non-crosslinked fibrin (0.9918 [0.9767-1.023] RU) vs. citrullinated crosslinked fibrin (0.7716 [0.6977-0.8493] RU). D: Citrullinated non-crosslinked fibrin (0.59 [0.5476-0.6662] RU) vs. citrullinated crosslinked fibrin (0.7716 [0.6977-0.8493] RU).

**Figure S4. Comparison of maximal clot firmness of different fibrin clots measured by viscoelastometry as described in Fig. 5.** A: Native non-crosslinked fibrin (0.9865 [0.9524-1.048] RU) vs. native crosslinked fibrin (1.091 [1.054-1.169] RU). B: Native non-crosslinked fibrin (0.9865 [0.9524-1.048] RU) vs. citrullinated non-crosslinked fibrin (0.9231 [0.5952-1.041] RU). C: Native non-crosslinked fibrin (0.9865 [0.9524-1.048] RU) vs. citrullinated crosslinked fibrin (1.268 [0.958-1.422] RU). D: Citrullinated non-crosslinked fibrin (0.9231 [0.5952-1.041] RU) vs. citrullinated crosslinked fibrin (1.268 [0.958-1.422] RU).

**Figure S5. Comparison of the storage modulus (G’, panels A-C) and the loss modulus (G”, panels D-F) of different fibrin clots measured by oscillatory rheometry as described in Fig. 4.** A: Native non-crosslinked fibrin (90.65 [74.14-123.00] Pa) vs. citrullinated non-crosslinked fibrin (65.23 [43.51-70.62] Pa). B: Native non-crosslinked fibrin (90.65 [74.14-123.00] Pa) vs. native crosslinked fibrin (273.9 [267.2-316.4] Pa). C: Citrullinated non-crosslinked fibrin (65.23 [43.51-70.62] Pa) vs. citrullinated crosslinked fibrin (209.3 [160.8-263.9] Pa). D: Native non-crosslinked fibrin (9.65 [6.04-12.31] Pa) vs. citrullinated non-crosslinked fibrin (7.16 [5.008-7.918] Pa). E: Native non-crosslinked fibrin (9.65 [6.04-12.31] Pa) vs. native crosslinked fibrin (19.46 [19.12-20.36] Pa). F: Citrullinated non-crosslinked fibrin (7.16 [5.008-7.918] Pa) vs. citrullinated crosslinked fibrin (14.20 [12.65-17.65] Pa).

**Figure S6. Comparison of the maximal bearable strain (*γ_max_*, RU, panels A-C) and the critical shear stress (*τ_0_*, Pa, panels D-F) of different fibrin clots measured by oscillatory rheometry as described in Fig. 4.** A: Native non-crosslinked fibrin (3.445 [1.89-3.78] RU) vs. citrullinated non-crosslinked fibrin (2.93 [1.76-3.59] RU). B: Native non-crosslinked fibrin (3.445 [1.89-3.78] RU) vs. native crosslinked fibrin (1.795 [1.32-2.01] RU). C: Citrullinated non-crosslinked fibrin (2.93 [1.76-3.59] RU) vs. citrullinated crosslinked fibrin (3.66 [2.292-4.600] RU). D: Native non-crosslinked fibrin (125.2 [93.12-145.6] Pa) vs. citrullinated non-crosslinked fibrin (94.93 [56.89-117.4] Pa). E: Native non-crosslinked fibrin (125.2 [93.12-145.6] Pa) vs. native crosslinked fibrin (729.3 [695.7-812.1] Pa). F: Citrullinated non-crosslinked fibrin (94.93 [56.89-117.4] Pa) vs. citrullinated crosslinked fibrin (586.9 [447.4-849.8] Pa).

**Figure S7. Comparison of the loss tangent (*G”/G’*, RU) of different fibrin clots measured by oscillatory rheometry as described in Fig. 4.** A: Native non-crosslinked fibrin (0.098 [0.092-0.107] RU) vs. citrullinated non-crosslinked fibrin (0.113 [0.1095-0.1187] RU). B: Native non-crosslinked fibrin (0.098 [0.092-0.107] RU) vs. native crosslinked fibrin (0.0685 [0.063-0.074] RU). C: Citrullinated non-crosslinked fibrin (0.113 [0.1095-0.1187] RU) vs. citrullinated crosslinked fibrin (0.068 [0.06525-0.078] RU).

**Table S3. Catalogue numbers and LOT numbers or specific activities of commercial enzymes and substrates used in the study.**

| **enzyme** | **manufacturer** | **Cat No.** | **LOT No./Batch No.** | **specific activity** |
| --- | --- | --- | --- | --- |
| Bovine thrombin | SERVA electrophoresis GmbH | 36402 |  | 1200 IU/mg |
| rFXIIIA2 | Zedira | T027 | 4223aT027 | 2475 U/mg |
| Peptidyl-arginine-deiminase 4 | Merck Sigma | SAE0086 |  | ≥200 U/mg |
| Recombinant tissue-type plasminogen activator (tPA) | Technoclone | TC41072 |  | ≥ 300 000 IU/mg |
|  |  |  |  |  |
| **substrate** |  |  |  |  |
| Human fibrinogen | Merck Millipore | 341578 | 4385541 |  |
| Spectrozyme-PL | Biomedica Diagnostics | 251L | 140919 |  |
